# Berberine-induced TFEB deacetylation by SIRT1 promotes autophagy in peritoneal macrophages

**DOI:** 10.18632/aging.202566

**Published:** 2021-02-26

**Authors:** Yinghong Zheng, Jiayuan Kou, Pengyu Wang, Ting Ye, Zitong Wang, Ziyu Gao, Lin Cong, Manman Li, Bowen Dong, Wei Yang, Quanfeng Li, Hong Li, Rui Wang, Liming Yang

**Affiliations:** 1 Department of Pathophysiology, Key Laboratory of Cardiovascular Pathophysiology, Harbin Medical University, Harbin 150081, China; 2 Yangpu Hospital, Tongji University, Shanghai 200090, China; 3 Department of Pathophysiology, Harbin Medical University (Daqing), Daqing 163319, China

**Keywords:** TFEB, SIRT1, deacetylation, peritoneal macrophage, berberine

## Abstract

Atherosclerosis is a chronic inflammatory disease that commonly affects the elderly and is characterized by vascular damage, macrophage infiltration, and plaque formation. Moreover, it increases the risk of cardiovascular disease. The pathogenesis of atherosclerosis involves an interplay between macrophage autophagy and apoptosis. A recently discovered transcription factor, transcription factor EB (TFEB) is known to activate autophagy in macrophages. Sirtuin deacetylase 1 (SIRT1), a nicotinamide adenine dinucleotide (NAD^+^)-dependent histone deacetylase, activates several transcription factors, including TFEB. We studied the effects of berberine on the NAD^+^ synthesis pathway and interactions between SIRT1 and TFEB. We also studied the effects of berberine-induced TFEB activation via SIRT1 on autophagy and apoptosis of peritoneal macrophages. We found that berberine promoted autophagy of peritoneal macrophages by activating SIRT1 via the NAD^+^ synthesis pathway and, in turn, promoting TFEB nuclear translocation and deacetylation. The functional regulation of SIRT1 and TFEB by berberine could be exploited as a potential therapeutic strategy for atherosclerosis.

## INTRODUCTION

In recent years, cardiovascular disease has emerged as the leading cause of human death. Atherosclerosis is a dominant risk factor for cardiovascular disease and shows an aging trend [1]. It is a complex pathophysiological process, with its development involving multiple cell types including macrophages [2]. Activated macrophages phagocytize foreign materials, release pro-inflammatory factors, recruit lymphocytes to strengthen immunity, protect the vasculature, and maintain tissue homeostasis [3]. Autophagy is a highly regulated cytoprotective process used by eukaryotic cells to degrade longevity proteins and damaged organelles [4]. In atherosclerosis, basal-level macrophage autophagy protects plaque cells against oxidative stress by degrading damaged intracellular material and reducing cell apoptosis [5]. In addition, macrophage autophagy restores cell metabolism by regulating the extension of cell membranes for the synthesis of autophagic lysosomes [6]. Macrophage apoptosis is a prominent feature of all stages of atherosclerosis [7]. Basal-level macrophage autophagy in atherosclerotic plaques contributes to plaque stability. However, excessive apoptosis of macrophages occurring in advanced atherosclerotic plaques causes necrosis, inflammation, and plaque instability and detachment [8].

Acetylation and deacetylation of N-terminal lysine residues in histone tails are catalyzed by histone acetyl transferases (HATs) and histone deacetylases (HDACs), respectively. This epigenetic modification allows proper nucleosome assembly and chromatin packaging and activate or inhibit gene transcription [9]. Sirtuin deacetylase 1 (SIRT1) is a class III HDAC, whose activity depends on cytoplasmic NAD^+^/NADH ratio [10].

Transcription factor EB (TFEB) is a member of the microphthalmia family of bHLH-LZ transcription factors (MiTF/TFE). TFEB regulates the expression of several lysosome and autophagy genes [11]. Under normal physiological conditions, TFEB is localized to cytoplasm. In response to starvation or external stimuli, it is rapidly translocated to the nucleus to activate the expression of several downstream genes [12]. Furthermore, TFEB affects the progression of atherosclerosis by promoting the phagocytosis ability of macrophages in plaques by enhancing lysosomal bactericidal performance [13].

Berberine, an alkaloid extracted from plants, is a traditional Chinese medicine used in several Asian countries for the past 1,400 years. Berberine-induced autophagy protects cardiac cells from the harmful effects of ischemia–reperfusion injury by accelerating energy circulation [14]. Furthermore, it prevents cardiac hypertrophy by reducing cardiomyocyte apoptosis [15]. Its anti-inflammatory and good lipid regulation properties make it a potential therapeutic candidate for atherosclerosis [16].

We studied the effects of berberine-induced TFEB activation via SIRT1 on autophagy and apoptosis in peritoneal macrophages and whether it protected against atherosclerosis.

## RESULTS

### Berberine-induced autophagy in peritoneal macrophages occurs earlier than apoptosis

**Figure 1:**
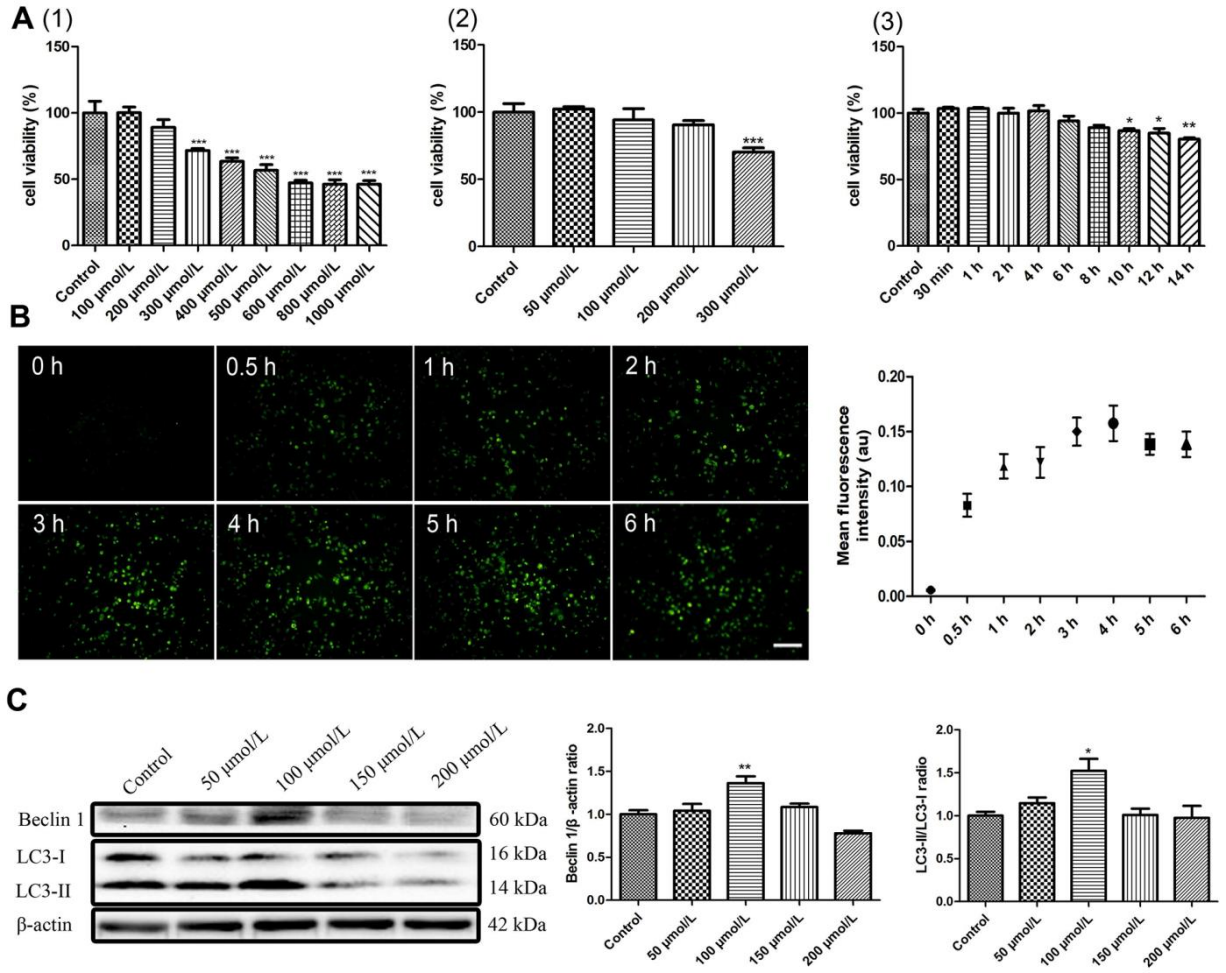
**Autophagy parameter selection of berberine.** (**A**) Effects of berberine on the viability of peritoneal macrophages. (1) Different concentrations of berberine (100–1000 μmol/L, 6 h). (2) Different concentrations of berberine (50–300 μmol/L, 6 h). (3) Different exposure times to berberine (100 μmol/L). Cell viability was analyzed by CCK-8 assay. Data was analyzed by one-way ANOVA with Tukey HSD post-hoc test (vs. Control group). (**B**) Intracellular accumulation of berberine (100 μmol/L) was detected in peritoneal macrophages (scale bar, 0.1 mm). (**C**) The expression of LC3 I, LC3 II, and Beclin 1 was analyzed by western blotting after treatment with different concentrations of berberine for 6 h. Quantification of LC3 II/LC3 I ratio and Beclin 1 is shown. Data was analyzed by one-way ANOVA with Tukey HSD post-hoc test (vs. Control group). All values are expressed as mean ± SD (error bars) of three independent experiments. *n* = 3; ^*^p < 0.05, ^**^p < 0.01, and ^***^p < 0.001 versus control.

**Figure 2:**
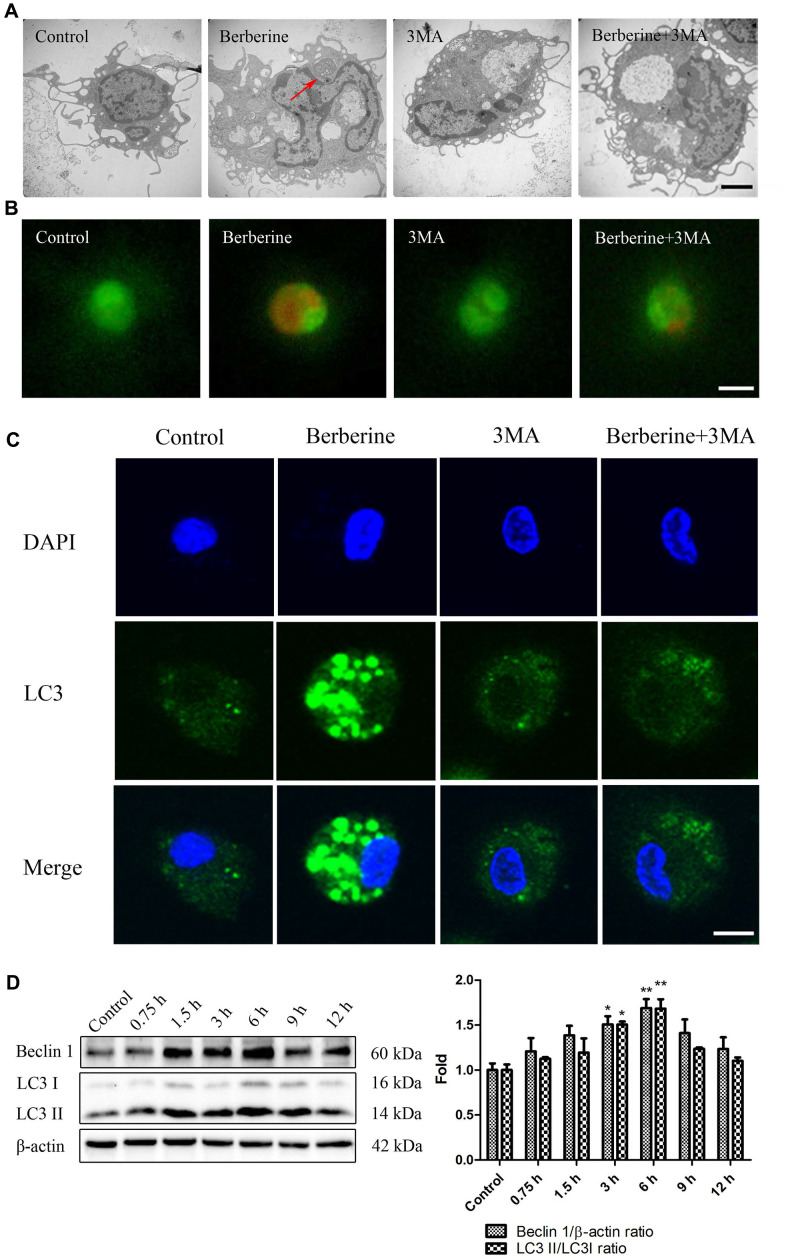
**Berberine triggers autophagy in peritoneal macrophages.** (**A**) Ultrastructural changes in untreated (control), 3MA only, and berberine-treated with or without 3MA peritoneal macrophages were observed by TEM at 6 h after berberine treatment. Red arrows indicate autophagosomes. Scale bar, 2 μm. (**B**) Peritoneal macrophages in control, 3MA only, and berberine-treated with or without 3MA groups incubated with acridine orange (50 μM) for 30 min. Scale bar, 50 μm. (**C**) Peritoneal macrophages stained with an anti-LC3B antibody and DAPI at 6 h after berberine treatment. Scale bar, 25 μm. (**D**) The expression of LC3 I, LC3 II, and Beclin 1 was analyzed by western blotting after treatment with berberine (100 μmol/L) for different time intervals. Data was analyzed by one-way ANOVA with Tukey HSD post-hoc test (vs. Control group). All values are expressed as mean ± SD (error bars) of three independent experiments. *n* = 3; ^*^p < 0.05, ^**^p < 0.01 versus control.

**Figure 3:**
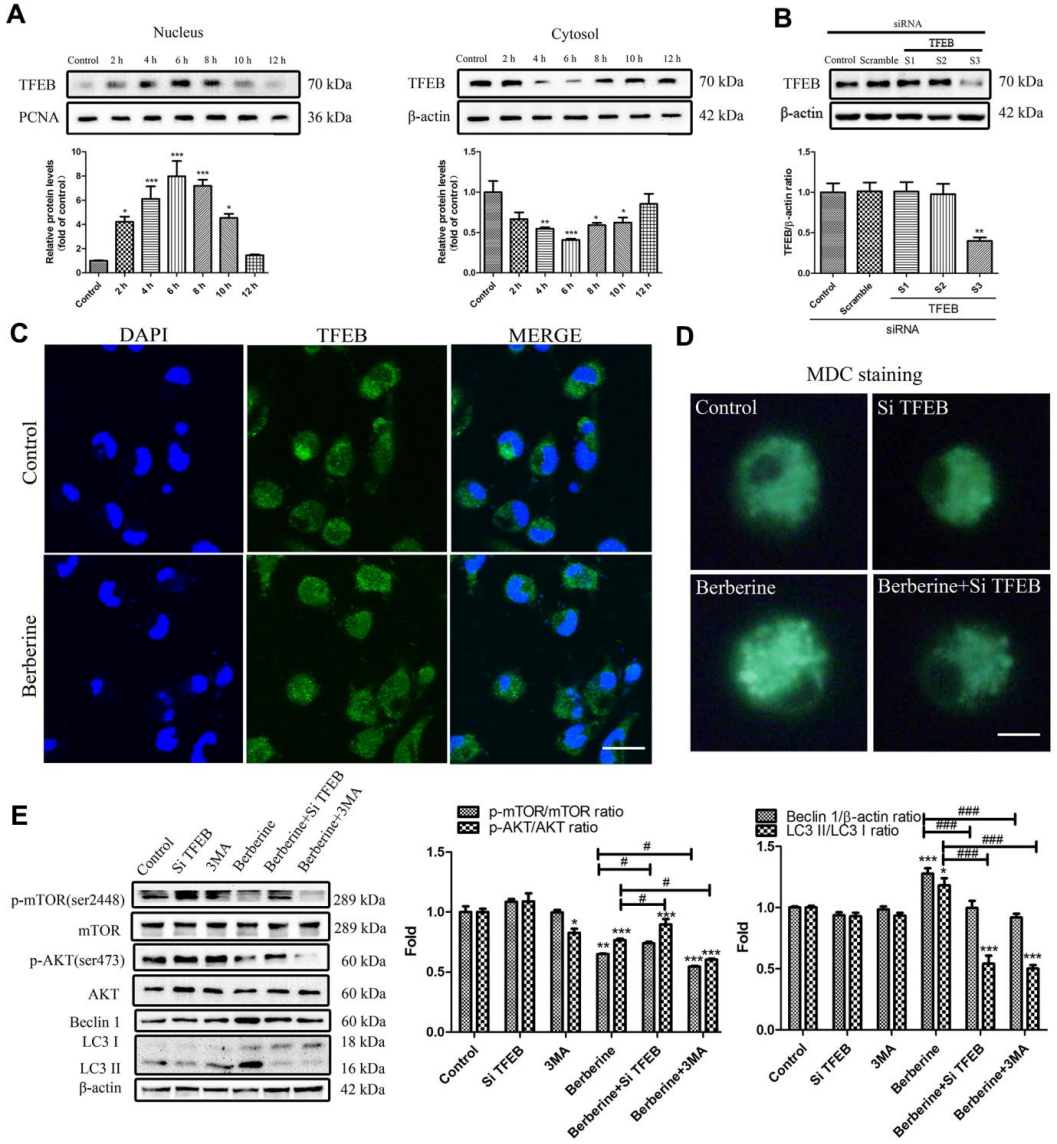
**Berberine-induced TFEB nuclear translocation promotes autophagy.** (**A**) The expression of TFEB in the nucleus and cytoplasm after treatment with berberine at different time points was detected by western blotting. Data was analyzed by one-way ANOVA with Tukey HSD post-hoc test (vs. Control group). (**B**) Representative western blots and quantification of TFEB following siRNA treatment. Data was analyzed by one-way ANOVA with Tukey HSD post-hoc test (vs. Control group). (**C**) Western blotting and immunofluorescence analysis of the effects on TFEB translocation on peritoneal macrophages at 6 h after treatment with berberine. Scale bar, 100 μm. (**D**) Formation of AVOs was detected by MDC staining after different treatments of peritoneal macrophages. Scale bar, 20 μm. (**E**) The expression of autophagy pathway proteins and autophagy-related proteins was detected by western blotting in peritoneal macrophages after different treatments. Data was analyzed by one-way ANOVA with Tukey HSD post-hoc test (vs. Control group). Analysis of variance and Student-Newman-Keuls post hoc tests were used to compare two group (vs. berberine group). All values are expressed as mean ± SD (error bars) of three independent experiments. *n* = 3; ^*^p < 0.05, ^**^p < 0.01, and ^***^p < 0.001 versus control. ^#^*p* <0.05, ^###^*p* < 0.001 versus berberine group.

A combination of berberine and ultrasound is known to induce macrophage autophagy [17]. To investigate the effects of berberine on autophagy and apoptosis, we first studied the intracellular accumulation of berberine and viability of macrophages after treatment with varying concentrations of berberine. Berberine has fluorescence and can be observed by fluorescence microscope after ingested by macrophages. Immunofluorescence studies revealed that berberine accumulated in macrophages in a time-dependent manner, reaching a peak at 3 to 4 h (Figure 1B). Figure 1A (1) (2) show that berberine was not toxic to cells in the range 0 to 200 μmol/L, whereas 300 μmol/L berberine resulted in cell death. To further explore the conditions of autophagy induced by berberine in peritoneal macrophages, we assessed the expression of autophagy-related proteins, namely Beclin 1, LC3 I, and LC3 II after treatment with different berberine concentrations. Macrophage autophagy was induced by 100 μmol/L berberine (Figure 1C). Moreover, at this concentration the cell viability remained unaffected until 10 h (Figure 1A (3)). To assess the optimal time required to induce cell autophagy by 100 μmol/L berberine, we studied the expression of Beclin 1, LC3 I, and LC3 II in peritoneal macrophages at different time points. Compared with the control group, the maximum expression was observed at 6 h post-berberine treatment (Figure 2D). To observe berberine-triggered autophagy-related intracellular changes, transmission electron microscopy (TEM), acridine orange staining, and immunofluorescence staining were used. Compared with the control group, double-membrane autophagosomes surrounding various damaged organelles were observed in the berberine group. Pre-treatment of cells with 3-methyladenine (3MA) reduced the formation of autophagosomes and acidic vesicle organelles (AVOs) and decreased the intracellular fluorescence intensity of LC3 (Figure 2A–2C). Moreover, it reduced the expression of Beclin 1 and inhibited the conversion of LC3 I to LC3 II (Figure 3E). Pre-treatment with chloroquine (CQ) in the berberine group resulted in further LC3 II and p62 accumulation compared with the cells treated with CQ alone (Supplementary Figure 2).

**Figure 4:**
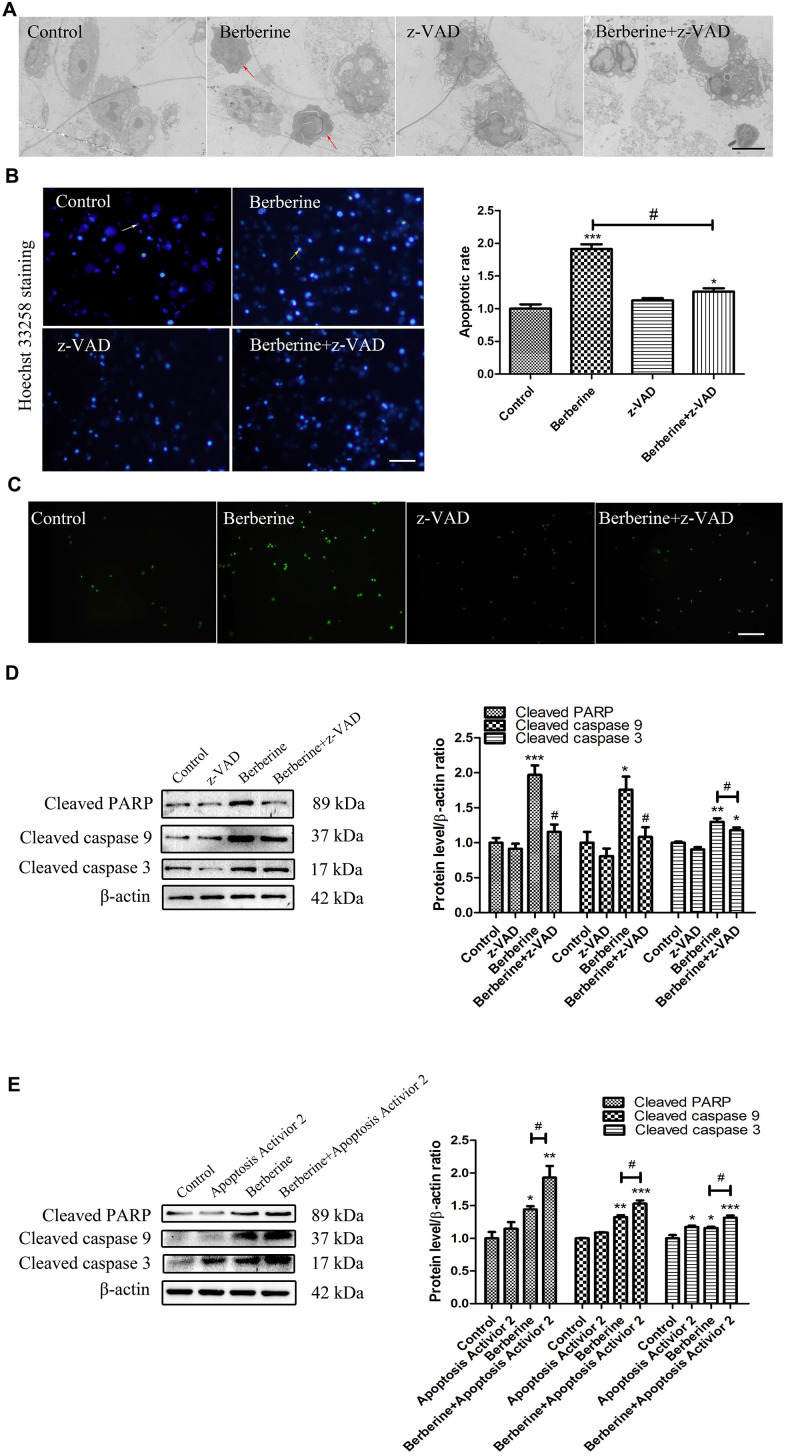
**Berberine induces apoptosis in peritoneal macrophages.** (**A**) Ultrastructural changes in untreated (control), z-VAD only, and berberine (100 μmol/L)-treated with or without z-VAD peritoneal macrophages observed by TEM at 10 h. Scale bar, 2 μm. Red arrows indicate that the cells showed typical apoptotic morphological changes, including the disappearance of cell microvilli, nuclear chromatin condensation on the nuclear envelope, and significant mitochondrial swelling with disappearing crista. (**B**) Apoptosis of peritoneal macrophages detected by Hoechst 33258 assay. Normal cells are indicated by uniform blue fluorescence (white arrow). Apoptotic cells appear as bright blue, fluorescent spots (yellow arrow, 0.1 mm). The percentage of apoptotic peritoneal macrophages ratio is shown. Scale bar, 500 μm (100 μmol/L berberine, 10 h incubation). Data was analyzed by one-way ANOVA with Tukey HSD post-hoc test (vs. Control group). Analysis of variance and Student-Newman-Keuls post hoc tests were used to compare two group (vs. berberine group). (**C**) Peritoneal macrophages in control, z-VAD only, and berberine-treated with or without z-VAD groups incubated with TUNEL reagent for 30 min. Scale bar, 500 μm (100 μmol/L berberine, 10 h incubation). (**D**) The effect of z-VAD on the protein content of cleaved PARP, cleaved caspase 9, and cleaved caspase 3 at 10 h after treatment with 100 μmol/L berberine. Quantification of proteins is shown. Data was analyzed by one-way ANOVA with Tukey HSD post-hoc test (vs. Control group). Analysis of variance and Student-Newman-Keuls post hoc tests were used to compare two group (vs. berberine group). (**E**) The effect of apoptosis activator 2 on the expression of cleaved PARP, cleaved caspase 9, and cleaved caspase 3 at 10 h after treatment with 100 μmol/L berberine. Quantification of proteins is shown. Data was analyzed by one-way ANOVA with Tukey HSD post-hoc test (vs. Control group). Analysis of variance and Student-Newman-Keuls post hoc tests were used to compare two group (vs. berberine group). All values are expressed as mean ± SD (error bars) of three independent experiments. *n* = 3; ^*^p < 0.05, ^**^p < 0.01, and ^***^p < 0.001 versus control. ^#^*p* < 0.05 versus berberine group.

We next examined the occurrence of apoptosis in macrophages treated with 100 μmol/L berberine for 10 h. Compared with the control group, the expression of apoptosis-related proteins, namely cleaved caspase 9, cleaved caspase 3, and cleaved PARP increased in berberine-treated cells. However, pre-treatment of cells with an apoptosis inhibitor z-VAD reversed this increase (Figure 4D). On the contrary, pre-treatment with Apoptosis Activator 2 enhanced the expression of apoptotic proteins induced by berberine (Figure 4E). TEM revealed ultrastructural changes, including typical apoptotic chromatin condensation on the interior surface of the nuclear envelope in macrophages treated with berberine for 10 h (Figure 4A). The number of Hoechst 33258-stained cells (blue) was higher in the berberine group than in the control group. Similarly, terminal deoxynucleotidyl transferase dUTP nick end labeling (TUNEL) staining revealed a higher number of bright green TUNEL-positive cells in the berberine group than in the control group. Pre-treatment of cultured macrophages with z-VAD reduced the number of apoptotic cells (Figure 4B, 4C). These results indicated that berberine induced macrophage apoptosis after 10 h.

**Figure 5:**
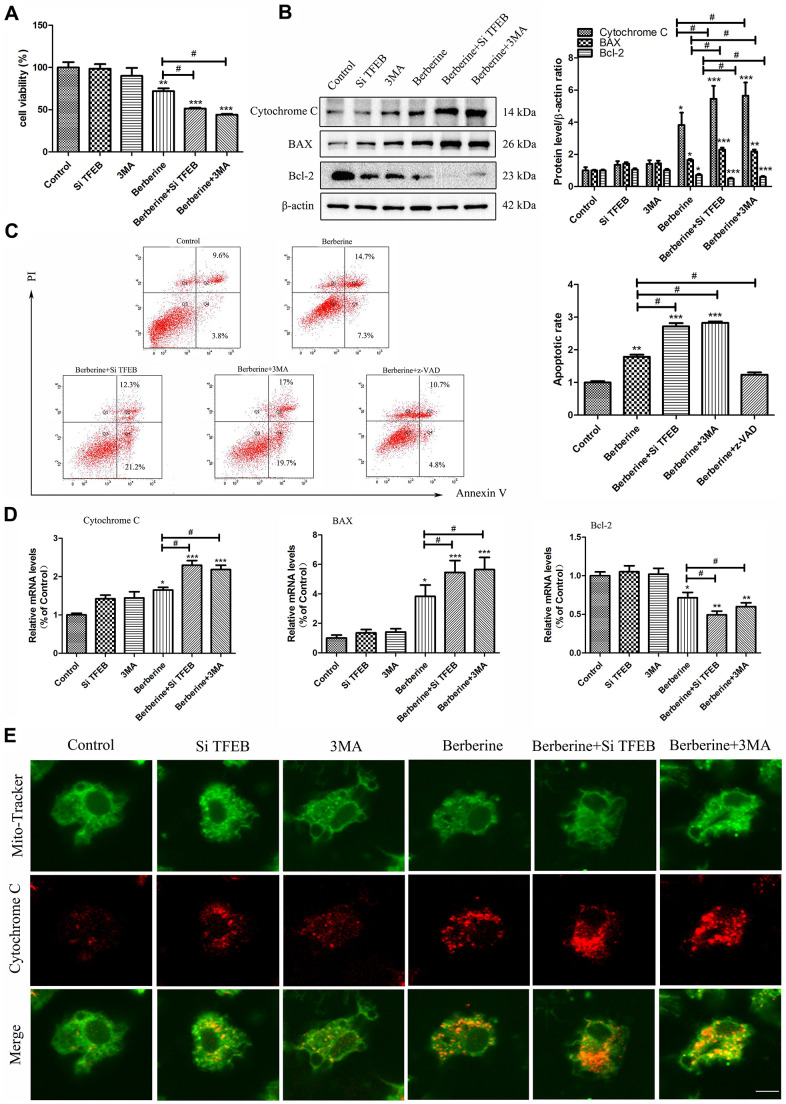
**TFEB nuclear translocation and autophagy induced by berberine inhibit apoptosis.** (**A**) CCK-8 assay was used to detect cell viability after different treatments (100 μmol/L berberine, 10 h incubation). Data was analyzed by one-way ANOVA with Tukey HSD post-hoc test (vs. Control group). Analysis of variance and Student-Newman-Keuls post hoc tests were used to compare two group (vs. berberine group). (**B**) The expression of apoptosis-related proteins BAX, Bcl-2, and cytochrome C was detected by western blotting following different treatments (100 μmol/L berberine, 10 h incubation). Data was analyzed by one-way ANOVA with Tukey HSD post-hoc test (vs. Control group). Analysis of variance and Student-Newman-Keuls post hoc tests were used to compare two group (vs. berberine group). (**C**) Changes in the number of apoptotic peritoneal macrophages were detected by flow cytometry (100 μmol/L berberine, 10 h incubation). Data was analyzed by one-way ANOVA with Tukey HSD post-hoc test (vs. Control group). Analysis of variance and Student-Newman-Keuls post hoc tests were used to compare two group (vs. berberine group). (**D**) The mRNA expression of apoptosis-related genes *BAX*, *Bcl-2,* and *cytochrome C* was detected by qRT-PCR after different treatments (100 μmol/L berberine, 10 h incubation). Data was analyzed by one-way ANOVA with Tukey HSD post-hoc test (vs. Control group). Analysis of variance and Student-Newman-Keuls post hoc tests were used to compare two group (vs. berberine group). (**E**) Changes in the level of released cytochrome C in peritoneal macrophages were detected using MitoTracker Green and anti-cytochrome C immunofluorescence staining. Scale bar, 20 μm (100 μmol/L berberine, 10 h incubation). All values are expressed as mean ± SD (error bars) of three independent experiments. *n* = 3; ^*^p < 0.05, ^**^p < 0.01, and ^***^p < 0.001 versus control. ^#^*p* < 0.05 versus berberine group.

Co-incubation of macrophages with berberine and 3MA for 10 h increased the number of apoptotic cells (Figure 5A, 5C), as evident from increased protein and mRNA expression of pro-apoptotic factor BAX. Moreover, the treatment reduced the expression of anti-apoptotic factor Bcl-2 and enhanced the accumulation of released cytochrome C (Figure 5B, 5D, 5E). These results demonstrated that inhibition of autophagy promoted cell apoptosis in peritoneal macrophages.

Based on these results, we conclude that berberine induced autophagy earlier (6 h) than apoptosis (10 h) in peritoneal macrophages. Treatment with 3MA reduced berberine-induced autophagy and promoted berberine-induced apoptosis.

### Berberine induces autophagy by inhibiting the PI3K/AKT/mTOR signaling pathway

PI3K/AKT is one of the mTOR signaling pathways that is known to suppress autophagy [18]. To explore the molecular mechanism of berberine-induced autophagy, we analyzed the phosphorylation levels of AKT and mammalian target of rapamycin (mTOR) (p-AKT and p-mTOR) at different time points following berberine treatment. As shown in Supplementary Figure 1A, berberine reduced p-AKT and p-mTOR levels, with minimum levels observed at 6 h that matched with the protein levels of Beclin 1, LC3 I, and LC3 II. To further study the crosstalk between the PI3K/AKT/mTOR signaling pathway and berberine-induced autophagy, cells in the berberine group were treated with or without selective inhibitors of class I PI3K (LY294002), AKT (triciribine), or mTOR (rapamycin, Rapa). Triciribine suppresses the phosphorylation of AKT, whereas Rapa inhibits the phosphorylation of mTOR. LY294002, triciribine, and Rapa enhanced the expression of Beclin 1 and increased the LC3 II/I ratio following berberine treatment (Supplementary Figure 1B–1D). In contrast, treatment with PI3K agonist insulin growth factor (IGF-1) reversed these results (Supplementary Figure 1E). Collectively, these results indicated that berberine induced autophagy in peritoneal macrophages by inhibiting the PI3K/AKT/mTOR signaling pathway.

### Berberine activates autophagy by targeting TFEB nuclear translocation

TFEB, a recently discovered transcription factor, controls the expression of genes involved in the formation of autophagic lysosomes, and the synthesis of lysosomal-related autophagy pathways. To study the crosstalk between TFEB and berberine-induced autophagy, we checked TFEB protein level in whole cell lysate and the effect of berberine to phosphorylation level of TFEB. Then we explored the expression of TFEB in both nucleus and cytosol following berberine treatment for different time intervals. We found that With the increase of berberine incubation time, the expression of TFEB in the whole cell did not change, but the phosphorylation level of TFEB decreased gradually at 0-6 hours, peaked at 6h, and gradually recovered after that (Supplementary Figure 3). Cell fractionation data showed that berberine induced the translocation of TFEB to the nucleus, with maximum translocation occurring at 6 h. The cytosolic TFEB levels recovered after 6 h (Figure 3A). Immunofluorescence data supported these results, which showed that TFEB was localized to the nucleus in the berberine group (Figure 3C). To further study the relationship between TFEB and autophagy, we knocked down *TFEB* by siRNA (*siTFEB)* (Figure 3B). Next, peritoneal macrophages were treated with berberine and with or without *siTFEB*, and the expression of autophagy-related proteins was assessed by western blotting. The results revealed that knocked down TFEB promoted the phosphorylation of AKT and mTOR and suppressed the expression of Beclin 1 and LC3 II/LC3 I (Figure 3E). To further study the effect of TFEB on autophagosomes following berberine treatment, we stained the cells with monodansylcadaverine (MDC), a fluorescent marker for autophagic vacuoles. The silencing of *TFEB* reduced the number of bright green vesicles observed in the berberine group (Figure 3D).

### Berberine-induced TFEB nuclear translocation inhibits apoptosis

Macrophage apoptosis in advanced plaques contributes to the pathogenesis of atherosclerosis by destabilizing the plaques. Thus, preventing macrophage apoptosis could be a therapeutic strategy. To explore the relationship between TFEB nuclear translocation and apoptosis following berberine treatment in peritoneal macrophages, we checked the cell viability using the CCK-8 assay. Compared with the cells in the berberine only group, *TFEB* silencing reduced the cell viability (Figure 5A). To further assess the effect of TFEB nuclear translocation on apoptosis, the expression of apoptotic proteins was detected by western blotting. Compared with the berberine group, the expression of pro-apoptotic factor BAX and apoptosis-related proteins, such as cytochrome C, increased following pre-treatment with *siTFEB*, whereas that of anti-apoptotic factor Bcl-2 decreased (Figure 5B). Similar results were observed for the mRNA levels of these proteins (Figure 5D). Pre-treatment with *siTFEB* increased the accumulation of berberine-induced apoptotic cells (Figure 5C). Further, immunofluorescence data revealed that pre-treatment with *siTFEB* promoted the berberine-induced release of cytochrome C (Figure 5E).

### Berberine promotes the formation of SIRT1/TFEB immune complex

**Figure 6:**
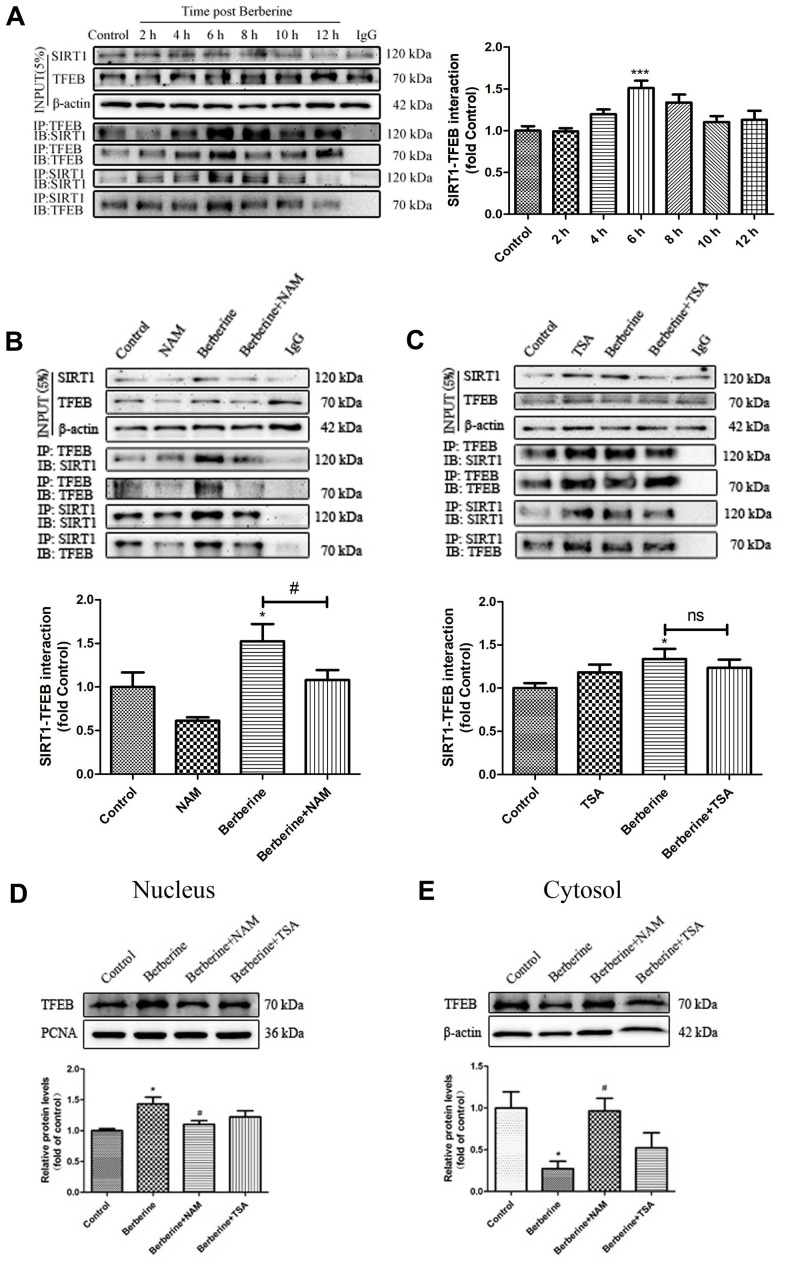
**Berberine promotes the formation of SIRT1/TFEB complex.** (**A**) Changes in the expression of SIRT1/TFEB complex in peritoneal macrophages at different incubation time points after treatment with 100 μmol/L berberine. Data was analyzed by one-way ANOVA with Tukey HSD post-hoc test (vs. Control group). (**B**) The effect of NAM on SIRT1/TFEB complex expression after treatment with 100 μmol/L berberine for 6 h. Data was analyzed by one-way ANOVA with Tukey HSD post-hoc test (vs. Control group). Analysis of variance and Student-Newman-Keuls post hoc tests were used to compare two group (vs. berberine group). (**C**) The effect of TSA on SIRT1/TFEB complex after treatment with 100 μmol/L berberine for 6h. Data was analyzed by one-way ANOVA with Tukey HSD post-hoc test (vs. Control group). Analysis of variance and Student-Newman-Keuls post hoc tests were used to compare two group (vs. berberine group). (**D**, **E**) The effects of NAM and TSA on TFEB translocation in peritoneal macrophages at 6 h after treatment with 100 μmol/L berberine. Data was analyzed by one-way ANOVA with Tukey HSD post-hoc test (vs. Control group). Analysis of variance and Student-Newman-Keuls post hoc tests were used to compare two group (vs. berberine group). All values are expressed as mean ± SD (error bars) of three independent experiments. *n* = 3; ^*^p < 0.05 versus control. ^#^*p* < 0.05 versus berberine group.

We next studied the effect of berberine on the relationship between SIRT1 and TFEB. We observed that following the incubation with berberine, SIRT1 gradually bound to TFEB in peritoneal primary macrophages in a time-dependent manner, with maximum binding occurring at 6 h (Figure 6A). The formation of berberine-induced SIRT1/TFEB complex was inhibited after treatment with nicotinamide (NAM), a SIRT1 inhibitor. However, the addition of protease inhibitor trichostatin A (TSA) did not affect the formation of the SIRT1/TFEB complex (Figure 6B, 6C). Moreover, NAM inhibited berberine-induced TFEB nuclear translocation, whereas the addition of TSA did not affect the TFEB nuclear translocation (Figure 6D, 6E). These results confirmed that berberine promoted the formation of SIRT1/TFEB complex by inducing TFEB nuclear translocation.

### Berberine activates SIRT1 in peritoneal macrophages through the NAD^+^ synthesis pathway

**Figure 7:**
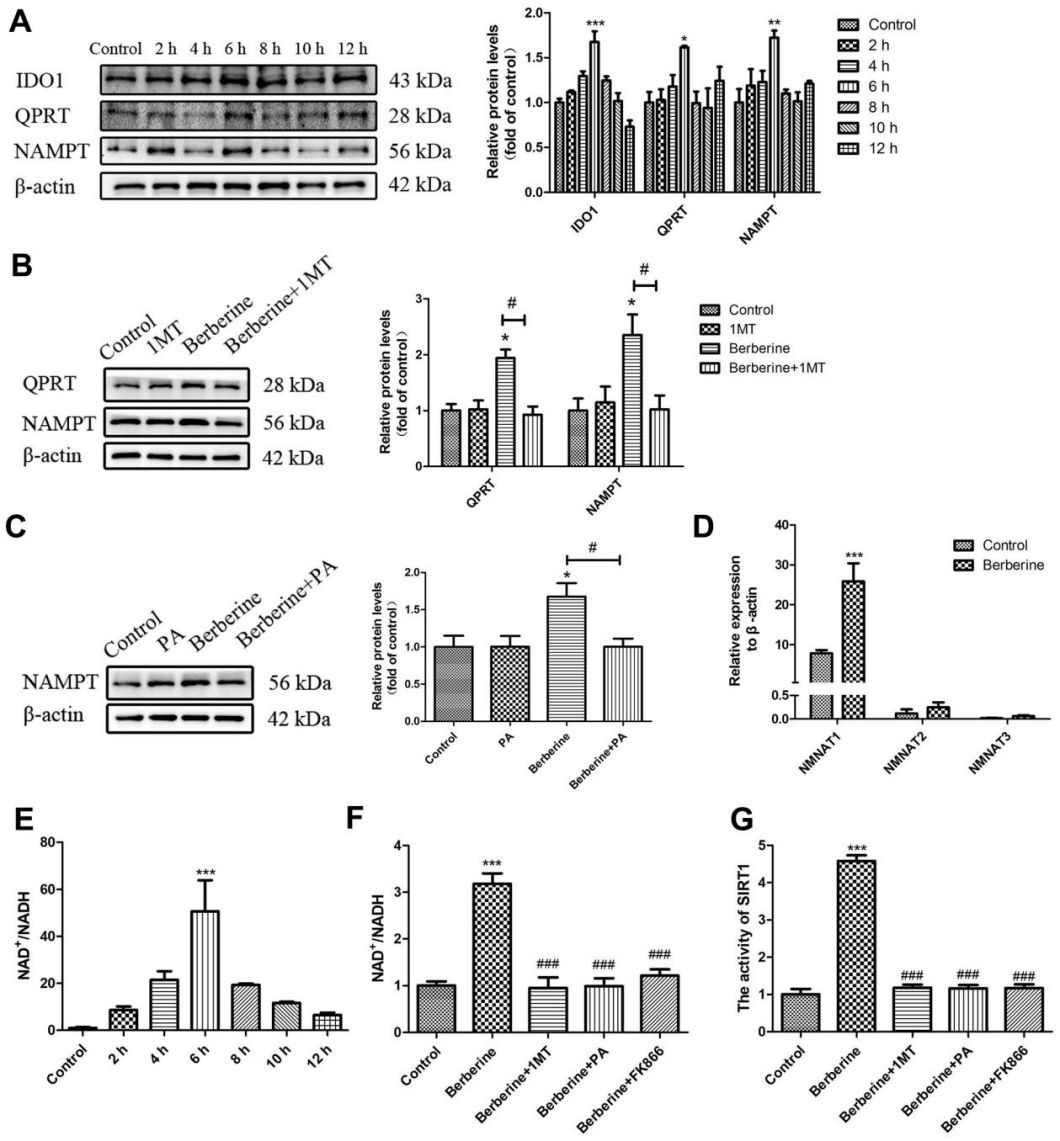
**Berberine activates SIRT1 via the NAD^+^ synthesis pathway.** (**A**) The relative protein expression of NAD^+^ synthesis pathway enzymes after treatment with 100 μmol/L berberine at different time points. Data was analyzed by one-way ANOVA with Tukey HSD post-hoc test (vs. Control group). (**B**) The effect of 1MT on the relative protein expression of NAD^+^ synthesis pathway enzymes after treatment with 100 μmol/L berberine. Data was analyzed by one-way ANOVA with Tukey HSD post-hoc test (vs. Control group). Analysis of variance and Student-Newman-Keuls post hoc tests were used to compare two group (vs. berberine group). (**C**) The effect of PA on the relative protein expression of NAMPT after treatment with 100 μmol/L berberine. Data was analyzed by one-way ANOVA with Tukey HSD post-hoc test (vs. Control group). Analysis of variance and Student-Newman-Keuls post hoc tests were used to compare two group (vs. berberine group). (**D**) The mRNA levels of NMNAT 1 to 3 after treatment with 100 μmol/L berberine. Data was analyzed by one-way ANOVA with Tukey HSD post-hoc test (vs. Control group). (**E**) NAD^+^/NADH level in peritoneal macrophages at different incubation time points after treatment with 100 μmol/L berberine. Data was analyzed by one-way ANOVA with Tukey HSD post-hoc test (vs. Control group). (**F**) The NAD^+^/NADH level in peritoneal macrophages following different treatments (100 μmol/L berberine, 6 h incubation). Data was analyzed by one-way ANOVA with Tukey HSD post-hoc test (vs. Control group). Analysis of variance and Student-Newman-Keuls post hoc tests were used to compare two group (vs. berberine group). (**G**) The activity of SIRT1 after different treatments (100 μmol/L berberine, 6 h incubation). Data was analyzed by one-way ANOVA with Tukey HSD post-hoc test (vs. Control group). Analysis of variance and Student-Newman-Keuls post hoc tests were used to compare two group (vs. berberine group). All values are expressed as mean ± SD (error bars) of three independent experiments. *n* = 3; ^*^p < 0.05, ^**^p < 0.01, and ^***^p < 0.001 versus control. ^#^*p* < 0.05, ^###^*p* < 0.001 versus berberine group.

We next studied the changes in the NAD^+^/NADH levels in primary peritoneal macrophages following incubation with berberine for different time intervals. Consistent with the time point results of SIRT1/TFEB complex formation (Figure 7E), changes in the NAD^+^/NADH level were time dependent, with a peak at 6 h. In addition, levels of proteins related to NAD^+^ synthesis pathways, such as indoleamine-2, 3-dioxygenase 1 (IDO1), quinolinate phosphoribosyl transferase (QPRT), and nicotinamide phosphoribosyltransferase (NAMPT), showed the same trend after berberine addition (Figure 7A). Compared with the berberine group, the expression of QPRT and NAMPT reduced after treatment with IDO1 inhibitor 1MT (Figure 7B). And also the expression of NAMPT reduced after treatment with QPRT inhibitor PA (Figure 7C). The results of quantitative reverse transcription polymerase chain reaction (qRT-PCR) showed that treatment with berberine for 6 h increased the mRNA levels of nicotinamide mononucleotide adenosyltransferase 1 (NMNAT1), whereas it had no major effect on the mRNA levels of NMNAT2 and NMNAT3 (Figure 7D). Treatment with 1MT, PA, or NAMPT inhibitor FK866 decreased the NAD^+^/NADH levels and reduced the berberine-induced SIRT1 activity (Figure 7F, 7G). These results proved that berberine increased the level of SIRT1 in primary peritoneal macrophages through the NAD^+^ synthesis pathway.

### Berberine-induced nuclear TFEB deacetylation is depended on SIRT1

**Figure 8:**
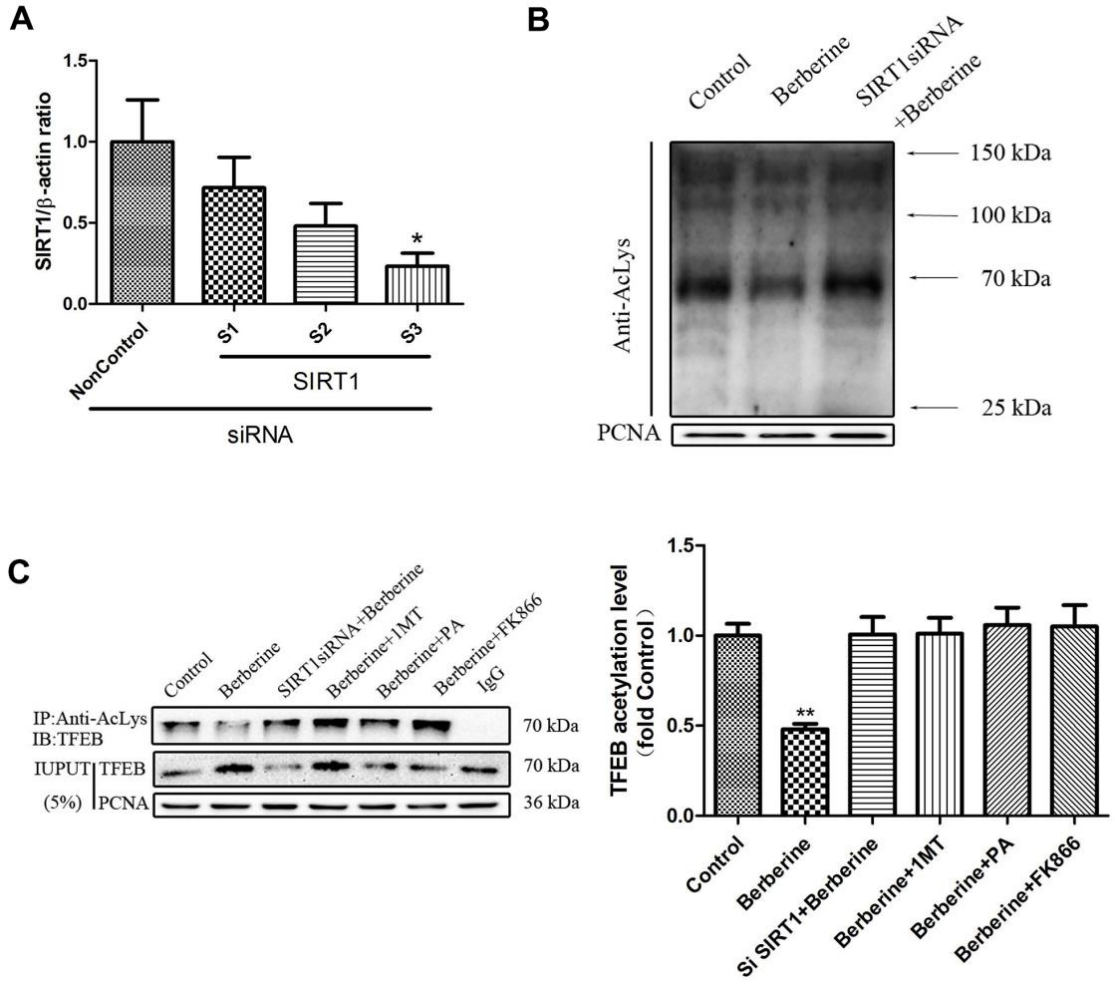
**Berberine deacetylates TFEB by activating SIRT1.** (**A**) The mRNA level of SIRT1 following siRNA treatment. Data was analyzed by one-way ANOVA with Tukey HSD post-hoc test (vs. Control group). (**B**) Acetylation levels in primary peritoneal macrophages after different treatments. Data was analyzed by one-way ANOVA with Tukey HSD post-hoc test (vs. Control group). (**C**) Acetylation level of TFEB in the nucleus of primary peritoneal macrophages after different treatments. All values are expressed as mean ± SD (error bars) of three independent experiments. *n* = 3; ^*^p < 0.05, ^**^p < 0.01 versus control.

The degree of acetylation of proteins in the nucleus reduced after treatment with berberine (Figure 8B). Treatment with *siSIRT1* (Figure 8A) reversed this effect (Figure 8B). Berberine reduced TFEB acetylation in the nucleus. Furthermore, the levels of acetylated TFEB in the nucleus increased after the addition of NAD^+^ synthesis pathway inhibitor or *siSIRT1* (Figure 8C, Supplementary Figure 4). These results proved that berberine promoted TFEB deacetylation in the nucleus by activating SIRT1.

## DISCUSSION

Macrophages maintain tissue homeostasis, promote tissue repair, and resist pathogens [19]. Early atherosclerosis lesions are characterized by damage to the vascular endothelium, and infiltration of monocytes under the epithelium and their differentiation into macrophages [20]. Because macrophages regulate lipid metabolism and inflammatory reactions, their number and function in plaques affect the development of atherosclerosis [21].

Berberine is an alkaloid extracted from plants. Although it is commonly dissolved in dimethylsulfoxide (DMSO) when used as an experimental drug, we found that water-soluble berberine easily entered and accumulated inside macrophages *in vitro*.

Autophagy and apoptosis are involved in the pathogenesis of several diseases. Autophagy is an intracellular quality control process that selectively degrades damaged cellular components to maintain internal homeostasis, thus contributing to cell survival. Moreover, it acts as a cytoprotective stress response mechanism to several disease conditions, with its inhibition leading to the accumulation of toxic proteins and dysfunctional organelles, resulting in oxidative stress, DNA damage, and genomic instability [22, 23]. The degree of autophagy affects the development of atherosclerosis. Basal-level macrophage autophagy exerts a protective effect and maintains homeostasis in the initial stages [24]. However, excessive autophagy causes the death of smooth muscle cells, plaque instability, and acute clinical events [25]. Berberine has been reported to protect cardiomyocytes against ischemia–reperfusion injury by inhibiting autophagy activation [26]. However, we show that berberine induced autophagy in peritoneal macrophages. In our previous study, berberine alone (under optimized conditions) did not induce autophagy in THP-1 macrophages [17], indicating that berberine-induced autophagy could be related to its concentration or cell type. The expression of mTOR, the primary regulator of autophagy, is activated by starvation, growth factors, and cellular stress factors. The PI3K/AKT survival pathway, present upstream of mTOR, inhibits mTOR activity [27]. We showed that berberine-induced autophagy suppressed the PI3K/AKT/mTOR signaling pathway.

The therapeutic use of berberine is based on its ability to induce both autophagy and apoptosis. For example, it exerts its anti-tumor effect by inducing apoptosis of pancreatic cancer cells [28]. The consequence of macrophage apoptosis on plaque formation and stability varies in different stages of atherosclerosis. Rapid recruitment of macrophages to an early atherosclerotic plaque efficiently clears apoptotic macrophages, resulting in limited local inflammation and diminished focal growth of lesions [29]. However, with the development of atherosclerosis, defective phagocytic clearance of apoptotic macrophages and vascular smooth muscle cells causes their accumulation and generates a pro-inflammatory response. Consequently, a necrotic core is formed that promotes further inflammation, and plaque instability and shedding [30]. Thus, macrophage apoptosis delays the progression of early atherosclerotic plaques, whereas it accelerates plaque formation in advanced stages.

In this study, we investigated the intervention of berberine in early atherosclerotic plaque. Our results showed that berberine could induce macrophages apoptosis after incubation for 10 hours, which occurred after autophagy in 6 hours. We found that berberine could promote autophagy and then apoptosis in peritoneal macrophages, which is beneficial to the intervention of the early pathological process of atherosclerosis. In addition, we used autophagy inhibitor 3MA in 6 hours, which increased apoptosis in Figure 5. It shows that autophagy and apoptosis of peritoneal macrophages induced by berberine are antagonistic to each other.

TFEB is a member of the microphthalmia family of bHLH-LZ transcription factors (MiTF/TFE) [31]. In response to starvation or other external stimuli, cytosolic TFEB is dephosphorylated and translocated to the nucleus, where it is deacetylated by SIRT1 to activate downstream lysosome and autophagy-related genes [32]. Several studies have reported the therapeutic use of TFEB in fatty liver, diabetes, and cardiac hypertrophy by regulating autophagy [33–35]. Moreover, TFEB aggravates apoptosis by negatively regulating autophagy [36]. We showed that TFEB promoted berberine-induced autophagy in peritoneal macrophages. Berberine-induced TFEB nuclear translocation confirmed that autophagy occurred earlier than apoptosis. Moreover, berberine induced autophagy through activated TFEB to inhibit apoptosis and promote macrophage survival, thus increasing plaque stability. Isotopic labeling and metabolic analysis in macrophages have shown NAD^+^ to regulate immune functions [37]. Cytoplasmic NAD^+^ is used as a co-substrate by several enzymes, including poly (ADP-ribose) polymerase (PARPs), cyclic ADP-ribose synthase, and SIRTs to form NAM, which is then converted to NAD^+^ by a recovery pathway initiated by NAMPT and NMNAT [38].

NAD^+^ homeostasis is often disrupted in senescence and metabolic diseases, where its increased consumption or reduced synthesis depletes NAD^+^ storage. Moreover, activation of PARPs or cADPR CD38 in immune cells depletes NAD^+^ [39, 40].

SIRT1 is a NAD^+^-dependent histone deacetylase that deacetylates several histone and non-histone proteins [41–43]. The activity of SIRT1 depends on the ratio of NAD^+^/NADH in the cytoplasm. Isotope tracing showed that intracellular NAD^+^ is primarily derived from tryptophan-canine urine metabolism [44]. Berberine activated SIRT1 in peritoneal macrophages by increasing the expression of key enzymes of NAD^+^ synthesis pathways, namely IDO1, QPRT, and NAMPT with a consequent increase in the NAD^+^/NADH ratio.

SIRT1 is known to deacetylate several transcription factors [45]. It enhances TFEB transcription by interacting with the TFEB immune complex, directly deacetylating lysine 116 [46]. During starvation, SIRT1 replaces the bromine domain protein BRD4, thereby removing the TFEB-inhibiting effect of BRD4 and activating TFEB to induce lysosomal synthesis and activate autophagy [47]. We showed the relationship between berberine-induced SIRT1 activity, nuclear translocation of TFEB followed by the formation of TFEB–SIRT1 complex, and TFEB deacetylation. NAM, a SIRT1 inhibitor, effectively inhibited TFEB nuclear translocation and prevented the formation of SIRT1/TFEB immune complex, suggesting that SIRT1 regulated berberine-induced TFEB nuclear translocation in peritoneal macrophages. Interestingly, MPB, a novel berberine derivative, also induced TFEB nuclear translocation by activating JNK and AMPK in macrophages. Therefore, it is also worth noting that, there were other pathways involved in the regulation of berberine induced TFEB nuclear translocation. Liu et al have shown that MPB switched on TFEB nuclear translocation by coupling 2 parallel signaling pathways. MPB-triggered JNK activation led to 14-3-3d released from TFEB, which, in turn, caused TFEB nuclear translocation. In addition, Liu et al have confirmed that AMPK mTOR signaling is required for MPB-induced TFEB nuclear translocation. MPB induced AMPK activation and subsequent inhibition of mechanistic target of rapamycin activity, which also contributed to TFEB nuclear translocation. MPB triggers K63-linked polyubiquitination of TAK1 at lysine 158, which, in turn, causes TAK1-mediated JNK and AMPK activation. MPB-induced JNK activation results in 14-3-3 released from TFEB, and MPB-induced AMPK activation suppresses mTOR activity, inducing TFEB nuclear translocation [48]. In addition, inhibitors of NAD^+^ synthesis pathway key enzymes or *siSIRT1* reduced nuclear TFEB deacetylation, indicating that TFEB deacetylation is dependent on the NAD^+^ synthesis pathway that regulates SIRT1 activity.

Therefore, we believe that SIRT1 and its upstream enzymes could serve as potential targets for the anti-atherogenic effects of TFEB.

**Figure 9:**
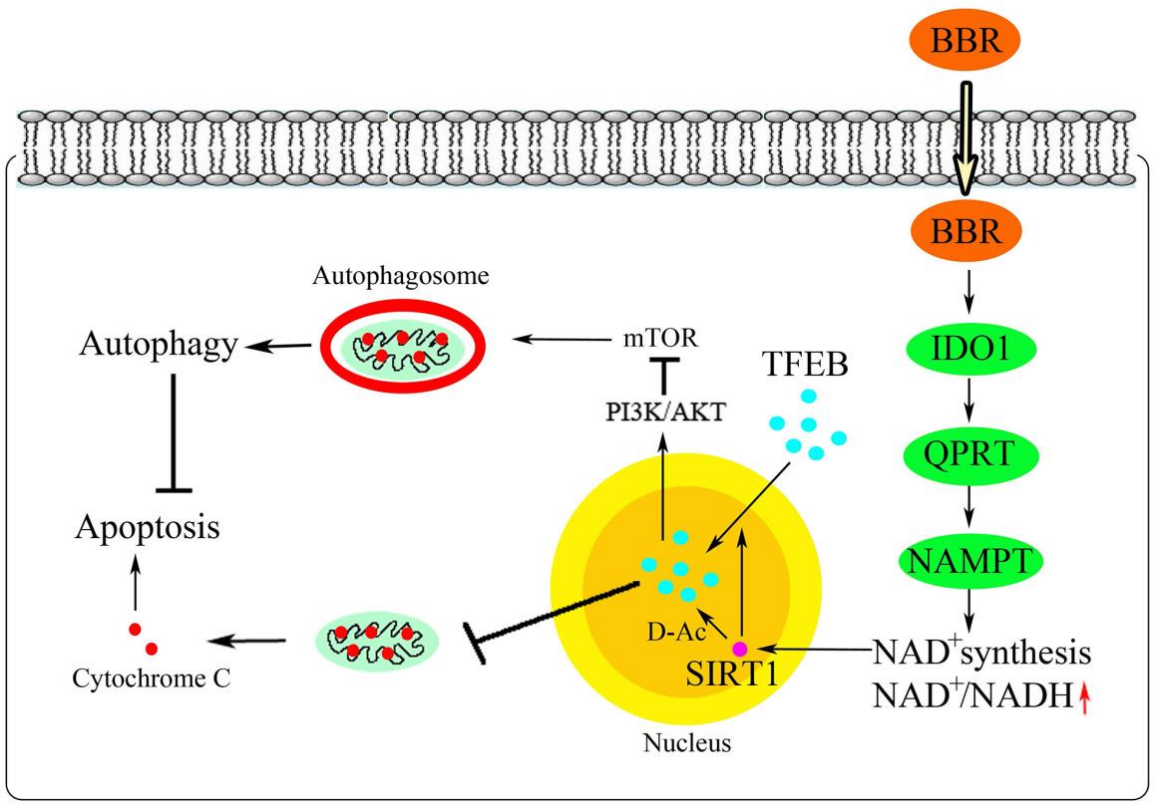
**Schematic illustration of the proposed mechanism.**

In summary, berberine activated SIRT1 through the NAD^+^ synthesis pathway to promote TFEB nuclear translocation, form SIRT1–TFEB immune complex, and deacetylate TFEB. These events subsequently triggered autophagy in peritoneal macrophages and inhibited apoptosis (Figure 9). Our data provide a new possibility for the treatment of atherosclerosis using berberine.

## MATERIALS AND METHODS

### Cell culture and animals

Peritoneal macrophages were extracted from C57BL/6 male mice following injection with 2 mL of 3% thioglycolate broth medium for 72 h. The cells were cultured in RPMI 1640 medium (HyClone; Logan, UT, USA) containing 10% fetal bovine serum (FBS, HyClone), 20 μg/mL penicillin, 20 μg/mL streptomycin (Sigma-Aldrich; St. Louis, MO, USA) for 24 h and maintained at 37° C in a humidified incubator with 5% CO_2_. For experiments, cells were seeded at a density of 1.0 × 10^5^ cells/mL in a 35 mm Petri dish or a 96-well plate. Complete medium was used to prevent autophagy and death due to starvation.

Eight-week-old, healthy C57BL/6 male mice, weighing 20 to 25 kg, were used in the study. The mice were provided by the laboratory animal center of the Second Affiliated Hospital in Harbin Medical University. All animal experiments were conducted according to the national standards.

All animal experiments were approved by the Institutional Animal Care and Use Committee of Harbin Medical University (No. HMUIRB-2008-06).

### Reagents

Berberine was purchased from Chengdu Must Bio-Technology Co. (Chengdu, China) and was stored in ddH_2_O as a 4 mM stock solution at 4° C in dark. The control group received an equivalent volume of medium.

To perform different inhibitory analyses, 10 mM autophagy inhibitor 3-methyladenine (3MA; Sigma-Aldrich Co.), 50 μM of the autophagy inhibitor chloroquine (Sigma-Aldrich Co.), 20 μM apoptosis inhibitor z-VAD-FMK (Beyotime Biotechnology; Beijing, China), 2 μM caspase 3 activator Apoptosis Activator 2 (Selleck Chemicals; Houston, TX, USA), 10 mM SIRT1 inhibitor nicotinamide (NAM, Beyotime Biotechnology), 10 mM HDAC inhibitor trichostatin A (TSA, Beyotime Biotechnology), 200 μM IDO1 inhibitor 1-methyl-L-tryptophan (1MT, Sigma-Aldrich Co.), 200 μM QPRT inhibitor phthalic acid (PA, Sigma-Aldrich Co.), 10 μM NAMPT inhibitor FK866 (Beyotime Biotechnology), 5 μM PI3K inhibitor LY294002 (Selleck Chemicals), 5 μM PI3K agonist insulin-like growth factor-1 (IGF-1, R&D Systems, Inc., Minneapolis, USA), 5 μM AKT inhibitor triciribine (Selleck Chemicals), and 1 μM mTOR inhibitor rapamycin (Rapa, Selleck Chemicals) were used in combination with berberine.

### Cell viability assay

Cell viability was assessed using the CCK-8 assay (Beyotime Biotechnology). Macrophages were seeded at equal density in 96-well culture plates. The plates were carefully washed twice with phosphate-buffered saline (PBS). To each well of the plate, 100 μL of RPMI medium containing CCK-8 (in a ratio of 9:1 [v/v]) was added following different treatments. After incubation for 30 min at 37° C in dark, the absorption of each well at 450 nm was measured by a microplate reader (Varian Australia Pty. Ltd., Australia).

### Cellular uptake of berberine

Cells were incubated with 100 μmol/L berberine for 0 to 6 h at 37° C in dark. After repeated washes with PBS, intracellular berberine was observed using confocal laser scanning microscopy (CLSM) (LSM 510 Meta; Zeiss, Gottingen, Germany) (Figure 1B). The absorption spectrum and fluorescence emission spectrum of berberine (dissolved in ddH_2_O) have been described previously. The fluorescence intensity of berberine was analyzed using Zeiss CLSM software (ZEN 2009 Light Edition).

### Transmission electron microscopy

Following treatment with berberine for different time intervals, the cells were centrifuged and fixed with 2.5% glutaraldehyde at 4° C overnight. Next, cells were observed under a transmission electron microscope (JEM-1220, Japan).

### Hoechst 33258 assay

Hoechst 33258 staining was used to detect apoptosis of macrophages. After treatment with berberine for 10 h, macrophages were incubated with 5 μg/mL Hoechst 33258 dye for 5 min at 37° C in dark. After repeated washes with PBS, the cells were examined under a fluorescence microscope (Olympus IX81, Japan) using a filter with a 420 to 480 nm emission wavelength. The acquired images were subsequently processed using Image-Pro Plus software (Media Cybernetics; Rockville, MD, USA).

### Terminal dUTP nick end-labeling assay

Berberine-induced apoptosis of macrophages was detected using the *In Situ* Cell Death Detection Kit, POD (Roche; Mannheim, Germany) according to the manufacturer’s instructions and as described previously [49]. After the TUNEL assay, macrophages were observed under a fluorescence microscope (Olympus IX81) to count TUNEL-positive cells.

### Immunofluorescence staining

At indicated time points after berberine treatment, cells were washed with PBS and fixed with 4% paraformaldehyde for 30 min and permeabilized in 1% Triton X-100 for 20 min at room temperature. After washing with PBS, cells were blocked with 3% bovine serum albumin (BSA), followed by incubation with anti-LC3B, anti-TFEB, or anti-cytochrome C antibody (1:200) overnight at 4° C. Cells were washed with PBS and incubated with the corresponding secondary antibody at 37° C for 1 h in dark. After washing with PBS, cells were stained with DAPI (1:200) or 100 nmol/L MitoTracker Green for 5 min. The cells were again washed with PBS to remove the extra stain. Cells in all groups were observed by laser scanning confocal microscopy (LSCM; LSCM 510 Meta; Zeiss).

### MDC staining

MDC (Sigma-Aldrich) was used to observe autophagic vacuoles. After different treatments, cells were incubated with 50 μmol/mL MDC dye in dark for 30 min at 37° C. After washing with PBS, the cells were observed under a fluorescence microscope (IX-71, Olympus).

### Acridine orange staining

Acridine orange staining (Sigma-Aldrich) was used to observe AOVs. At 6 h after treatment with berberine, cells were incubated with 0.01% acridine orange for 30 min at 37° C in dark. After washing twice with PBS, cells were observed under a fluorescence microscope (IX-71, Olympus).

### Western blotting

After different treatments, radioimmunoprecipitation assay (RIPA) lysis buffer was used to extract total protein. The protein concentration was assessed using a bicinchoninic acid (BCA) kit (Beyotime Biotechnology). After denaturation, protein samples were separated on a sodium dodecyl sulfate-polyacrylamide gel (SDS-PAGE) and transferred onto a 0.45 μm PVDF membrane (Millipore; Schwalbach, Germany). After blocking with 5% low-fat milk diluted with Tris-buffered saline containing Tween 20 (TBST), the membrane was incubated with primary antibodies at 4° C overnight. After washing with TBST, the membranes were incubated with horseradish peroxidase (HRP)-conjugated secondary antibodies for 2 h at room temperature. After washing with TBST again, the immune complexes were detected using an enhanced chemiluminescence reagent. The protein bands were quantified using Quantity One software (Bio-Rad Laboratories; Hercules, CA, USA) and normalized to β-actin or PCNA.

Antibodies against mTOR (# 2983S), p-mTOR (# 5536S), AKT (# 4685S), p-AKT (# 4060S), TFEB (# 32361S), p-TFEB (#86843S), p62 (#16177S), cleaved caspase 9 (# 9509S), cleaved caspase 3 (# 9661S), cleaved PARP (# 9548S), cytochrome C (# 11940S), SIRT1 (# 8469S), IDO1 (# 51851S), and acetylated lysine (# 9441S) (1:1000) were purchased from Cell Signaling Technology (Boston, USA). Antibodies against BAX (OM122725) and Bcl-2 (OM241170) (1:1000) were purchased from Omnimabs (California, USA). Antibodies against Beclin 1 (ab62557) and NAMPT (ab45890) (1:1000) were purchased from Abcam (Burlingame, CA, USA). Antibody against QPRT (LS-C749352) (1:1000) was purchased from LifeSpan BioSciences (USA). Antibody against LC3B (L7543) (1:1000) was purchased from Sigma-Aldrich. Antibody against β-actin (60008-1-Ig) (1:1000) was purchased from Proteintech Group (Wuhan, China). HRP-conjugated secondary mouse (ZB-5305) and rabbit (ZB-5301) antibodies (1:5000) were purchased from Zhongshan Company (Beijing, China).

### Annexin V/PI flow cytometry

Annexin V-fluorescein isothiocyanate (FITC)/ propidium iodide (PI) Apoptosis Detection Kit (BD Pharmingen; Franklin Lakes, NJ, USA) was used to detect apoptosis according to the manufacturer’s instructions. After treatment with berberine for 10 h with or without pre-treatment with 3MA, z-VAD, or *siTFEB*, the cells were collected and incubated with 5 μL of Annexin V-FITC and 10 μL of PI for 15 min at room temperature in dark. After filtration, the cells were analyzed by fluorescence-activated cell sorting Calibur system within 1 h. Annexin V^+^/PI^-^ and Annexin V^+^/PI^+^ represented apoptotic cells in early and late phases, respectively. The data were analyzed using BD FACSDiva Software v7.0 (Becton-Dickinson, USA).

### Small interfering RNA transfection

Small interfering RNAs (siRNAs) was used to knockdown *TFEB* and *SIRT1* in macrophages. An irrelevant 21-nucleotide siRNA was used as negative control (GenePharma, Shanghai, China). The target sequences are listed in Table 1. The efficiency of transfection was confirmed using western blotting.

**Table 1:** Target sequences.

**TFEB (mouse)**	si#1: 5′-GAAAGACAATCACAACCTA-3′
	si#2: 5′-CCATGGCCATGCTACATAT-3′
	si#3: 5′-CCAAGAAGGATCTGGACTT-3′
**SIRT1 (mouse)**	si#1: 5′-UGAAAGAACUGUACCACA-3′
	si#2: 5′-GGAAGAUGAAAGUGAAAUUTT-3′
	si#3: 5′-UGUCAGAUAAGGAAGGAAATT-3′

### Quantitative reverse transcription polymerase chain reaction

Total RNAs were extracted using Trizol reagent (Invitrogen; Carlsbad, California, USA) and reverse-transcribed using the All-in-one cDNA Synthesis SuperMix Kit (Bimake, Houston, USA). Next, 1 μL of cDNA was amplified in a 2× SYBR Green qPCR Master Mix (Bimake) on ABI 7900HT Sequence Detection System (ABI Applied Biosystems; Foster City, CA). The primers used are listed in Table 2. Gene expression values were normalized against that of β-actin. Fold induction was calculated using the expression values of each gene at time 0 (uninfected) in peritoneal macrophages as a reference.

**Table 2:** Primers used.

Cytochrome C (mouse)	Forward: 5′-AAGGCATCACCTGGGGAGAG-3′
	Reverse: 5′-GGTCTGCCCTTTCTCCCTTCT-3′
Bcl-2 (mouse)	Forward: 5′-TCTTCGTCAGCTTCGACACCA-3′
	Reverse: 5′-TCACGACGGTAGCGACGAGAG-3′
BAX (mouse)	Forward: 5′-TTGCCCTCTTCTACTTTGCTAG-3′
	Reverse: 5′-CCATGATGGTTCTGATCAGCTC -3′
SIRT1 (mouse)	Forward: 5′-CGCTGTGGCAGATTGTTATTAA -3′
	Reverse: 5′-TTGATCTGAAGTCAGGAATCCC-3′
NMNAT1 (mouse)	Forward: 5′-GTGATGCGTACAAGAAGAAAGG-3′
	Reverse: 5′-TCTGAAGACTTTCCCACGTATC-3′
NMNAT2 (mouse)	Forward: 5′-GTCAAGTCGGCACCGTCTCATC-3′
	Reverse: 5′-CCGATCACAGGTGTCATGGAAGG-3′
NMNAT3 (mouse)	Forward: 5′-CAGCAAGACACCATCAGCCTCTG-3′
	Reverse: 5′-ACGCACACCAAGCCGAACTTC-3′
β-actin (mouse)	Forward: 5′-GTGCTATGTTGCTCTAGACTTCG-3′
	Reverse: 5′-ATGCCACAGGATTCCATACC-3′

### NAD^+^/NADH detection

To each group of cultured cells, 200 μL of NAD^+^/NADH extract pre-cooled on ice bath was added after the culture medium was discarded. Next, cells were gently beaten to promote cell lysis and centrifuged at 12,000 g at 4° C for 5 to 10 min. The supernatant was collected for later use. To obtain the NADH standard curve, a 10 mM NADH standard solution was diluted with NAD^+^/NADH extract into an appropriate concentration gradient. Ethanol dehydrogenase was diluted 45 times with reaction buffer. Next, 50 to 100 μL of samples to be measured was taken into a centrifuge tube and heated at 60° C in a water bath or on a PCR machine for 30 min to decompose NAD^+^. Next, 10 μL of a chromogenic solution in NAD^+^/NADH Detection Kit (Beyotime, China) was added to each well, mixed well, and incubated at 37° C in light for 30 min to form orange formazan. The absorbance was measured at 450 nm.

### SIRT1 activity

After the treatment of cells with SIRT1, YIJI lysate (Reagent A) was added. The cells were mixed and incubated in an ice tank for 15 min. Next, YIJI pre-cooled Reagent B was added and mixed well. The solution was centrifuged in a table centrifuge at 4° C for 10 min at 1,300 g. The supernatant was removed carefully and the pre-cooled YIJI cleaning solution (Reagent C) was added. The solution was centrifuged at 4° C for 10 min at 1,300 g. The supernatant was removed carefully and pre-cooled YIJI extract (Reagent D) was added and mixed well. The solution was transferred to a new pre-cooled 1.5 mL centrifuge, treated with ultrasound for 30 s, and incubated in an ice tank for 30 min. The solution was centrifuged at 4° C for 10 min at 16,000 g. The supernatant was transferred to a new pre-cooled 1.5 mL centrifuge tube. Next, 10 μL of supernatant was removed to quantitatively detect the proteins, and the enzyme reading was performed according to the instructions.

### Co-immunoprecipitation

After pre-treatment, cells were lysed with five times the volume of lysis buffer (25 mM Tris-HCl, 150 mM NaCl, 3 mM MgCl_2_, 1% Triton X-100, 0.5% NP-40, 1 mM DTT, 5% glycerin, 1% PI, pH 7.5) at 4° C for 2 h and centrifuged at 21,000 g for 30 min. The supernatant was measured and quantified with BCA Protein Assay Kit (Beyotime, China). The same amount of protein was incubated with the M2 affinity gel overnight at 4° C. The immunoprecipitate was washed with lysis buffer four times and eluted with SDS loading buffer. The samples were then subjected to SDS-PAGE.

### Acetylation level

After different treatments, native cytosolic and nuclear proteins were rapidly separated using the Minute Cytoplasmic and Nuclear Extraction Kit (Invent Biotechnologies). The medium was discarded and cells were washed in cold PBS once. The cells were harvested in a 1.5 mL microcentrifuge tube in suspension by low-speed centrifugation (500 g for 3 min). The cells were washed in cold PBS once. The cells were transferred to a 1.5 mL microcentrifuge tube and centrifuged at 500 g for 1 min. The supernatant was aspirated completely. Next, 100 μL of cytoplasmic extraction buffer was added to cell pellets, the tube was vortexed vigorously for 15 s, incubated on ice for 5 min, and again vortexed briefly. The cells were centrifuged for 5 min at 15000 g speed in a microcentrifuge at 4° C. The supernatant (cytosol fraction) was transferred to a fresh pre-chilled 1.5 mL tube. Next, 50 μL nuclear extraction buffer was added to the pellet. The pellet was vortexed vigorously for 15 s and incubated on ice for 1 min. This step was repeated four times. The nuclear extract was immediately transferred to a pre-chilled filter cartridge containing a collection tube and centrifuged at 14,000 to 16,000 g in a microcentrifuge for 30 s. The filter cartridge was discarded and the nuclear extract was stored at –80° C until use. An anti-acetyl lysine antibody was used to immunoprecipitate the protein complexes (see method 17), followed by western blotting for TFEB.

### Statistical analysis

All experiments were replicated at least thrice independently. The data were analyzed using one-way analysis of variance (ANOVA) and Student’s *t*-test. Results are presented as mean ± standard deviation (SD). A *p* < 0.05 was considered significant.

## Supplementary Material

Supplementary Figures
