# Supplementary material for: Berberine-induced TFEB deacetylation by SIRT1 promotes autophagy in peritoneal macrophages
Source: Aging (Albany NY). 2021 Feb 26;13(5):7096–119. doi: 10.18632/aging.202566 (PMC7993719; doi:10.18632/aging.202566)
Supplement: Supplementary Figures [file aging-13-202566-s001.pdf]

SUPPLEMENTARY FIGURES

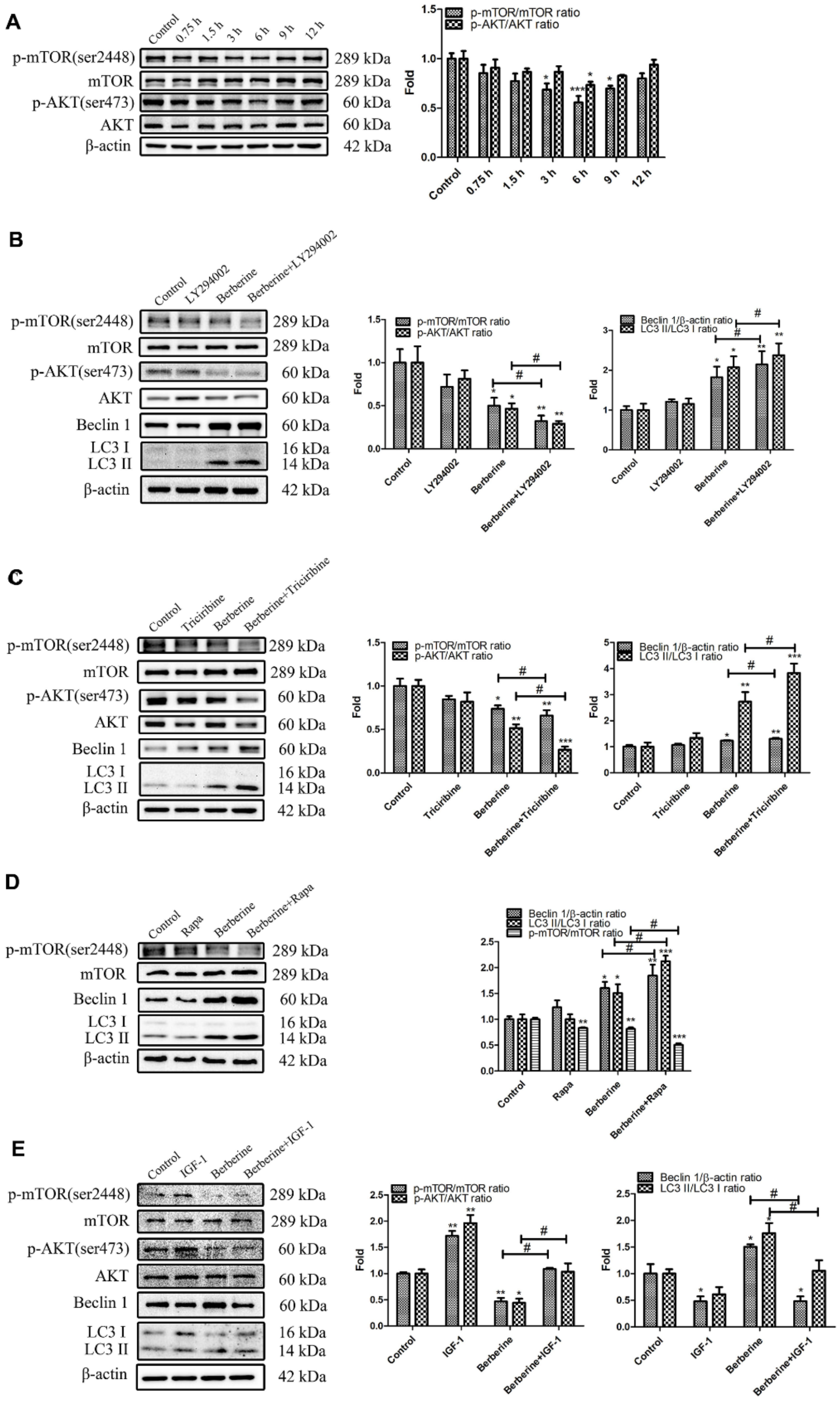

Supplementary Figure 1. The PI3K/AKT/mTOR signaling pathway is involved in berberine-induced autophagy in peritoneal macrophages. (**A**) The expression of mTOR, p-mTOR (Ser 2448), AKT, and p-AKT (Ser 473) was analyzed by western blotting at different time points after treatment with berberine. Quantification of the p-mTOR/mTOR ratio and p-AKT/AKT ratio is shown. Data was analyzed by one-way ANOVA with Tukey HSD post-hoc test (vs. Control group). Analysis of variance and Student-Newman-Keuls post hoc tests were used to compare two group (vs. berberine group). (**B**) The effect of LY294002 on the expression of mTOR, p-mTOR (Ser 2448), AKT, p-AKT (Ser 473), LC3 I, LC3 II, and Beclin 1 at 6 h after treatment with berberine. Quantification of proteins is shown. Data was analyzed by one-way ANOVA

with Tukey HSD post-hoc test (vs. Control group). Analysis of variance and Student-Newman-Keuls post hoc tests were used to compare two group (vs. berberine group). (**C**) The effect of triciribine on the expression of mTOR, p-mTOR (Ser 2448), AKT, p-AKT (Ser 473), LC3 I, LC3 II, and Beclin 1 at 6 h after treatment with berberine. Quantification of proteins is shown. Data was analyzed by one-way ANOVA with Tukey HSD post-hoc test (vs. Control group). Analysis of variance and Student-Newman-Keuls post hoc tests were used to compare two group (vs. berberine group). (**D**) The effect of rapamycin on the expression mTOR, p-mTOR (Ser 2448), LC3 I, LC3 II, and Beclin 1 at 6 h after treatment with berberine. Quantification of proteins is shown. Data was analyzed by one-way ANOVA with Tukey HSD post-hoc test (vs. Control group). Analysis of variance and Student-Newman-Keuls post hoc tests were used to compare two group (vs. berberine group). (**E**) The effect of IGF-1 on the expression of mTOR, p-mTOR (Ser 2448), AKT, p-AKT (Ser 473), LC3 I, LC3 II, and Beclin 1 at 6 h after treatment with berberine. Quantifications of proteins is shown. Data was analyzed by one-way ANOVA with Tukey HSD post-hoc test (vs. Control group). Analysis of variance and Student-Newman-Keuls post hoc tests were used to compare two group (vs. berberine group). All values are expressed as mean ± SD (error bars) of three independent experiments. $n = 3$; $^{*}p < 0.05$, $^{**}p < 0.01$, and $^{***}p < 0.001$ versus control. $^{\#}p < 0.05$ versus berberine group.

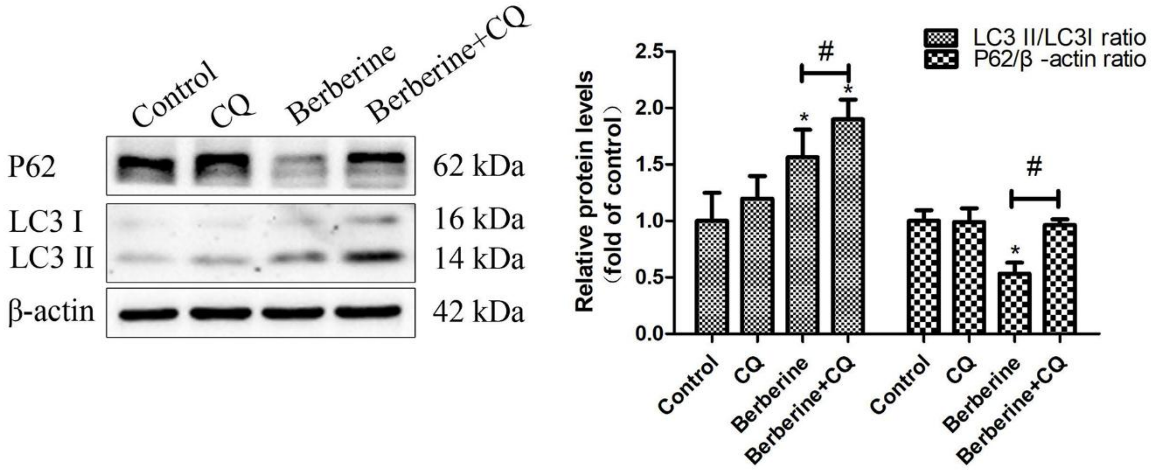

Supplementary Figure 2. The effect of CQ on autophagy induced by berberine. The expression levels of P62, LC3 I, LC3 II protein at 6 h after treating with berberine, and quantifications of the proteins above were shown. Data was analyzed by one-way ANOVA with Tukey HSD post-hoc test (vs. Control group). Analysis of variance and Student-Newman-Keuls post hoc tests were used to compare two group (vs. berberine group). All data are mean ± standard deviation. $n = 3$; $^{*}p < 0.05$ versus control. $^{\#}p < 0.05$ versus berberine group.

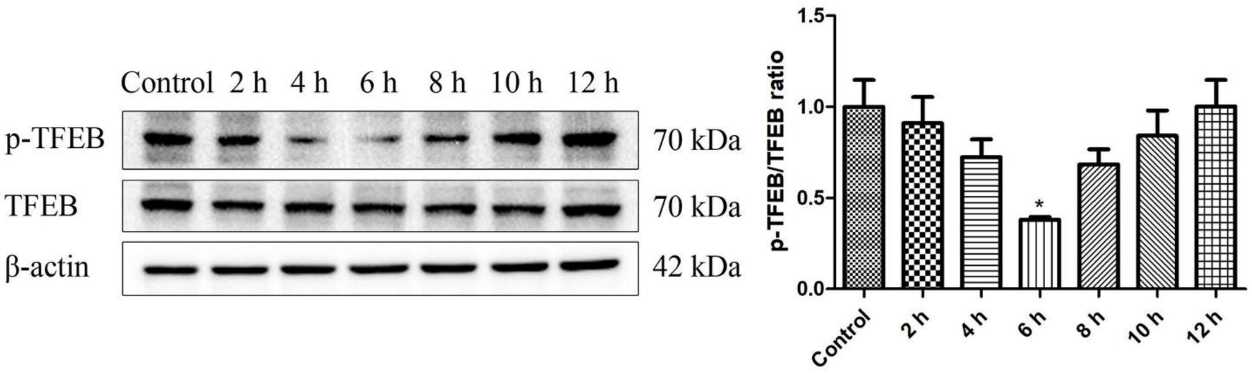

Supplementary Figure 3. Berberine-induced TFEB dephosphorylation in different time points. Data was analyzed by one-way ANOVA with Tukey HSD post-hoc test (vs. Control group). All data are mean ± standard deviation. $n = 3$; $^{*}p < 0.05$ versus control.

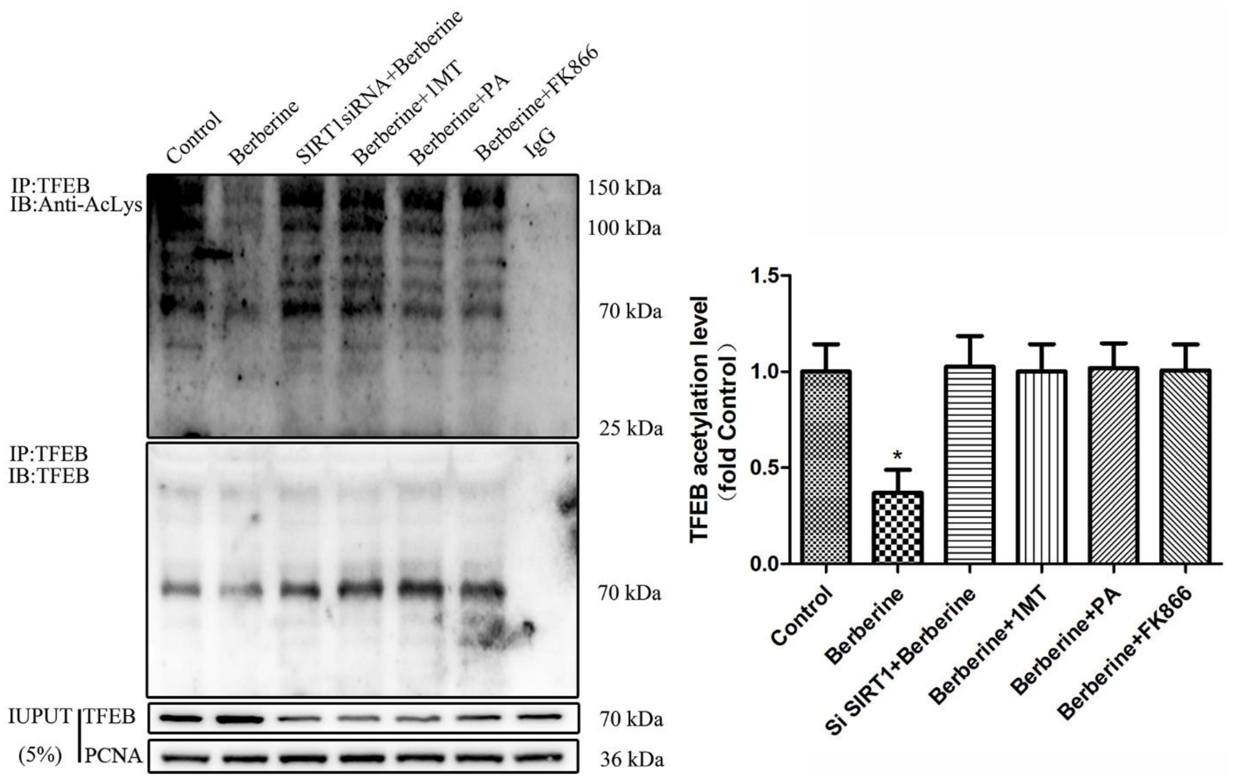

Supplementary Figure 4. TFEB acetylation level in different treatments for 6 h. Data was analyzed by one-way ANOVA with Tukey HSD post-hoc test (vs. Control group). All data are mean ± standard deviation. *n* = 3; *p < 0.05 versus control.
